# Disseminated *Nocardia farcinica* with multiple cerebral abscesses in a patient receiving induction immunosuppression for autoimmune hepatitis: case report

**DOI:** 10.3389/fmed.2026.1825538

**Published:** 2026-07-16

**Authors:** Akram Alnounou, Dimitar Bahariev, John Brown, Henry Zou, Harry Boamah

**Affiliations:** Western Michigan University Homer Stryker MD School of Medicine, Kalamazoo, MI, United States

**Keywords:** autoimmune hepatitis, disseminated nocardiosis, hemorrhagic brain abscess, immunosuppression, *Nocardia farcinica*, opportunistic infection, retroperitoneal abscess

## Abstract

**Background:**

Nocardiosis is an opportunistic infection caused by aerobic, Gram-positive actinomycetes of the genus *Nocardia* that most often affects patients with impaired cell-mediated immunity, including those receiving corticosteroids or other immunosuppressive therapies. Disseminated disease frequently involves the central nervous system, where a systematic review reported an overall case fatality rate of 22.8%, and *Nocardia farcinica* is the most commonly identified species. We report a case of disseminated *Nocardia farcinica* with multiple cerebral abscesses in a patient receiving induction immunosuppressive therapy for autoimmune hepatitis.

**Case report:**

A 55-year-old man with a history of autoimmune hepatitis and occupational soil exposure presented with 4–5 week history of progressive cognitive impairment, dysarthria, and right upper extremity weakness. Two months earlier, he had been treated with high-dose corticosteroids and azathioprine and prescribed trimethoprim-sulfamethoxazole (TMP-SMX) prophylaxis, which had been interrupted for an estimated 2–4 weeks before presentation during ongoing corticosteroid therapy. Upon admission, laboratory tests demonstrated neutrophil-predominant leukocytosis with lymphopenia, and liver function tests showed substantial improvement compared to his initial presentation of autoimmune hepatitis. Neuroimaging revealed multiple ring-enhancing intracerebral lesions with surrounding vasogenic edema. Stereotactic aspiration of a cerebral abscess and drainage of a concurrent retroperitoneal collection grew *Nocardia farcinica*, confirming disseminated nocardiosis. Antimicrobial therapy was transitioned to imipenem-cilastatin and TMP-SMX based on susceptibility testing. During infection management, azathioprine was paused, and prednisone was continued at a dose of 25 mg/day, followed by a biochemical autoimmune hepatitis flare requiring hepatology-guided monitoring. The patient demonstrated gradual neurologic improvement and was discharged to inpatient rehabilitation after an 18-day hospitalization.

**Conclusion:**

Disseminated nocardiosis should be considered for immunosuppressed patients with autoimmune hepatitis presenting with subacute neurological decline and ring-enhancing brain lesions, particularly during outpatient immunosuppression transitions when antimicrobial prophylaxis may be interrupted or incompletely reconciled. The patient demonstrated progressive neurological improvement and radiographic abscess reduction after 3 months of susceptibility-guided combination therapy, reinforcing the importance of early tissue diagnosis and neurosurgical intervention in this population.

## Introduction

Nocardiosis is an opportunistic infection caused by aerobic, Gram-positive actinomycetes of the genus *Nocardia* (1). *Nocardia* species are ubiquitous environmental organisms, and infection is most commonly acquired through inhalation, with less frequent cutaneous inoculation (1). The risk of developing nocardiosis is increased in patients with impaired cell-mediated immunity, including those receiving corticosteroids or other immunosuppressive therapies, organ transplant recipients, individuals with malignancy, and those with primary immune deficiencies (1, 2). The clinical presentation of nocardiosis may be subacute, and symptoms vary by organ involvement (1). Disseminated nocardiosis, characterized by the involvement of two or more non-contiguous organs, frequently involves the central nervous system (CNS), which is a clinically consequential site for infection (2). In a geographically unrestricted systematic review of 206 English-language reported cases of adult CNS nocardiosis, the overall case-fatality rate was 22.8%, with immunodeficient status independently associated with poor outcomes (3). *N. farcinica* is the most frequently identified species in CNS nocardiosis, accounting for approximately 40% of cases, and it is associated with higher reported rates of dissemination and intrinsic multidrug resistance than many other *Nocardia* species (3–5). Brain imaging is recommended for patients with suspected or confirmed invasive nocardiosis because CNS disease may be present even without neurological symptoms (2). Trimethoprim–sulfamethoxazole (TMP-SMX) is widely used for pneumocystis prophylaxis and is associated with a reduced risk of nocardiosis in solid organ transplant recipients; however, breakthrough nocardiosis can still occur despite prophylaxis (6). We report a case of disseminated *Nocardia farcinica* with multiple cerebral abscesses in a patient receiving induction immunosuppression for autoimmune hepatitis. Published reports of CNS nocardiosis associated with autoimmune hepatitis remain limited. Previous cases include severe *Nocardia asteroides* brain abscesses with ventriculitis in a patient receiving azathioprine and prednisolone (7), *N. farcinica* brain abscesses in patients receiving prednisolone for autoimmune hepatitis (8, 9), and disseminated *Nocardia otitidiscaviarum* presenting with ruptured intracranial mycotic aneurysm (10). The present case adds to this literature by documenting induction-phase immunosuppression for autoimmune hepatitis, suspected TMP-SMX prophylaxis interruption during the outpatient transition of steroid tapering, multiple hemorrhagic cerebral abscesses, microbiological confirmation of *N. farcinica* from two non-contiguous sites, and serial radiographic response to susceptibility-guided therapy.

## Case description

A 55-year-old right-handed man with a history of autoimmune hepatitis and occupational soil exposure presented to the emergency department with confusion, delayed responses, an inability to complete routine tasks, and progressive cognitive decline that had been ongoing for the last 4–5 weeks. He had been diagnosed with autoimmune hepatitis (AIH) 2 months prior to presentation, after a liver biopsy revealed marked portal inflammation, cholestasis, parenchymal collapse, and syncytial giant cell transformation. At the time, laboratory evaluation demonstrated hepatocellular injury consistent with active autoimmune hepatitis (AST 3242 U/L, ALT 4721 U/L, total bilirubin 19.4 mg/dL, and INR 2.0). For his AIH, he received intravenous methylprednisolone (20 mg every 8 h for 3 days) and was discharged on high-dose oral prednisone (20 mg three times daily), trimethoprim-sulfamethoxazole (TMP-SMX) prophylaxis (800–160 mg) three times weekly, and azathioprine 50 mg daily. Based on medication records, the cumulative corticosteroid exposure before the nocardiosis diagnosis was approximately 3,300 mg prednisone-equivalent, including intravenous methylprednisolone followed by oral prednisone up to 60 mg/day and later up to 25 mg/day. This exposure occurred during the induction phase of AIH therapy with concurrent azathioprine. One month prior to his current admission, the patient was hospitalized for a hyponatremia-induced generalized tonic–clonic seizure, and a non-contrast head computed tomography (CT) scan did not show any abnormalities. He was hospitalized again shortly afterward for steroid-induced psychosis and mild encephalopathy. During hepatology follow-up 1 week prior to the current admission, he reported that he had run out of azathioprine and TMP-SMX and was only taking prednisone, which was decreased to 25 mg daily with a tapering plan. He was also advised to restart azathioprine 50 mg daily and titrate in 25 mg increments to 100 mg daily, with liver elastography planned once liver enzymes normalized. A few days prior to the current admission, his spouse reported new-onset dysarthria, word-finding difficulties, right upper extremity weakness, imbalance, and drooling. On physical examination, the patient was lethargic but could be aroused, oriented only to person, with moderate dysarthria, non-fluent speech, impaired word retrieval, and an inconsistent ability to follow commands. The motor strength was 5/5 throughout, except for the right upper extremity, which was 4/5. The patient also exhibited impaired balance. The clinical timeline is summarized in Table 1.

**Table 1:** Clinical timeline: summary of key clinical events in a patient with autoimmune hepatitis who developed disseminated *Nocardia farcinica* with central nervous system involvement.

Timepoint	Clinical event
~2-month pre-admission (AIH diagnosis)	The liver biopsy established autoimmune hepatitis. The patient’s test results revealed an AST level of 3,242 U/L, ALT level of 4,721 U/L, total bilirubin of 19.4 mg/dL, and INR 2.0. Treatment included intravenous methylprednisolone for 3 days. The patient was then discharged with a regimen of prednisone 60 mg/day (divided), azathioprine 50 mg daily, and TMP-SMX prophylaxis three times weekly.
~4–5 weeks pre-admission	Progressive cognitive decline, dysarthria, and right upper extremity weakness began.
~1-month pre-admission	The patient was hospitalized for hyponatremia-associated generalized tonic–clonic seizure and later rehospitalized for steroid-induced psychosis and mild encephalopathy.
~1-week pre-admission (hepatology visit)	He had run out of azathioprine and TMP-SMX; prednisone was reduced to 25 mg daily with a tapering plan, and he was advised to restart azathioprine.
Hospital day 0 (presentation)	He presented with confusion, dysarthria, and right upper extremity weakness; had a witnessed generalized tonic–clonic seizure. A head CT scan showed multiple hemorrhagic intra-axial lesions. Started on levetiracetam plus empiric vancomycin, ceftriaxone, and metronidazole.
Hospital day 0 laboratory assessment	Admission WBC 11.4 × 10^9^/L with neutrophils 84%, ANC 9.5 × 10^9^/L, lymphocytes 4%, ALC 0.5 × 10^9^/L, monocytes 5%, and eosinophils 0%; AST 48 U/L, ALT 46 U/L, total bilirubin 2.9 mg/dL, and INR 1.1.
Hospital day 3	CT chest/abdomen/pelvis showed a left retroperitoneal collection. Brain MRI showed multiple ring-enhancing lesions with restricted diffusion and a thin right frontal epidural collection.
Early hospital course	Stereotactic aspiration of a frontal brain abscess and drainage of the retroperitoneal collection both grew *Nocardia farcinica*, confirming disseminated nocardiosis. Antibiotics were changed to imipenem-cilastatin plus TMP-SMX after susceptibility results.
Microbiologic diagnosis/targeted therapy transition	WBC normalized to 8.5 × 10^9^/L with ANC 7.1 × 10^9^/L and ALC 0.6 × 10^9^/L. Cerebral and retroperitoneal cultures confirmed *Nocardia farcinica*; therapy changed to imipenem-cilastatin plus TMP-SMX.
Immunosuppression modification/biochemical AIH flare	Azathioprine held, and prednisone continued at 25 mg/day during acute infection management. AST increased to 190 U/L and ALT to 220 U/L, while INR remained 1.1 and total bilirubin improved to 1.8 mg/dL; follow-up AST/ALT improved to 52/66 U/L.
Hospital days 12–18	PEG tube placed for nutrition and prolonged enteral antimicrobial therapy; neurologic status improved, and he was transferred in stable condition to acute inpatient rehabilitation on day 18.
~1 month after discharge	Follow-up MRI showed interval decrease in abscess size, decreased edema, and resolution of the extra-axial collection.
3-month follow-up	Recovered to approximately 80–90% of pre-illness baseline; dysarthria and expressive aphasia resolved; PEG removed; mild short-term memory deficits and residual right upper extremity numbness persisted. TMP-SMX was changed to linezolid because of rash.

### Diagnostic assessment

The laboratory evaluation on admission revealed leukocytosis (11.4 × 10^9^/L) with neutrophil predominance and lymphopenia: neutrophils 84%, absolute neutrophil count 9.5 × 10^9^/L, lymphocytes 4%, absolute lymphocyte count 0.5 × 10^9^/L, monocytes 5%, and eosinophils 0%. Additional admission studies showed hyponatremia (133 mmol/L), AST 48 U/L, ALT 46 U/L, total bilirubin 2.9 mg/dL, and INR 1.1. At the time of microbiologic diagnosis and transition to targeted therapy, WBC had normalized to 8.5 × 10^9^/L, with persistent neutrophil predominance and lymphopenia: absolute neutrophil count 7.1 × 10^9^/L and absolute lymphocyte count 0.6 × 10^9^/L. Non-contrast head CT showed multiple bilateral supratentorial hemorrhagic intra-axial lesions, the largest (1.5 cm) in the left thalamus, accompanied by surrounding vasogenic edema and a left-to-right midline shift of 2–3 mm (Supplementary Figure 1). The CT scan of the chest/abdomen/pelvis with contrast on hospital day 3 demonstrated a 1.6-cm left retroperitoneal collection, mild pulmonary interstitial edema with trace pleural effusions, a small pericardial effusion, and trace pelvic ascites. No pulmonary nodules, cavitary lesions, focal consolidations, lung abscess, tree-in-bud opacities, or other radiographic findings suggesting pulmonary nocardiosis were identified. No cutaneous nodules, ulcers, cellulitis, abscesses, draining wounds, or traumatic inoculation sites were documented on admission or during hospitalization. The MRI confirmed multiple ring-enhancing lesions with restricted diffusion, consistent with intracerebral abscesses, involving the left thalamus and basal ganglia, bilateral parietal-occipital regions, right frontoparietal cortex, and left frontal cortex, along with a thin right frontal epidural collection suggesting the possibility of empyema (Figure 1). Given the patient’s immunosuppression and the combination of ring enhancement, restricted diffusion, and hemorrhagic components, the differential diagnosis included pyogenic abscesses (including *Nocardia*), fungal infection, mycobacterial infection, toxoplasmosis, and metastatic disease. The evaluation did not identify an alternative systemic infectious source or a competing diagnosis, as blood cultures, HIV testing, serum cryptococcal antigen testing, QuantiFERON-TB testing, and toxoplasma serologies were negative. Echocardiography showed no vegetation. Tissue sampling was performed to obtain a definitive diagnosis. Frameless stereotactic aspiration of a superficial left frontal abscess and image-guided drainage of a left retroperitoneal collection both yielded *Nocardia farcinica* on culture, confirming disseminated nocardiosis. Species-level identification was confirmed by 16S rRNA gene sequencing performed by the clinical microbiology laboratory. Antimicrobial susceptibility testing demonstrated sensitivity to amoxicillin-clavulanate, imipenem, amikacin, TMP-SMX, and linezolid, with ciprofloxacin resistance and intermediate susceptibility to minocycline and ceftriaxone (MIC 32 μg/mL).

**Figure 1:**
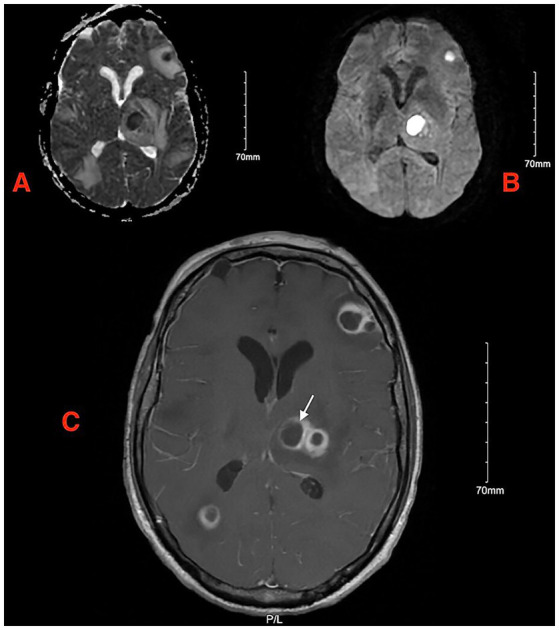
Brain MRI. Representative axial magnetic resonance images, including apparent diffusion coefficient mapping **(A)**, diffusion-weighted imaging **(B)**, and post-contrast T1-weighted imaging **(C)**, demonstrate multiple supratentorial intra-axial lesions with central diffusion restriction, peripheral enhancement, and prominent perilesional edema, most consistent with intracranial abscesses. A representative 2.5 × 2.0 cm left thalamic lesion is indicated by the arrow, with an associated mass effect and approximately 3 mm left-to-right midline shift.

### Therapeutic intervention

In the emergency department, he was started on levetiracetam for a witnessed generalized tonic–clonic seizure and received empiric vancomycin, ceftriaxone, and metronidazole. After microbiological confirmation and susceptibility testing, infectious disease consultants changed the regimen to imipenem-cilastatin 500 mg intravenously every 6 h and TMP-SMX suspension (40/200 mg per 5 mL, 40 mL dose) every 6 h via nasogastric tube; renal function was normal at treatment initiation. The hospital course was complicated by a brief focal seizure and transient metabolic derangements that were medically managed. On hospital day 12, gastroenterology was consulted for percutaneous endoscopic gastrostomy (PEG) tube placement to support nutrition and prolonged enteral antimicrobial therapy in the setting of dysphagia. Vascular surgery was recommended for tunneled central access. The infectious diseases team recommended intravenous imipenem-cilastatin and TMP-SMX for 3 months or until radiographic resolution of abscesses, followed by oral TMP-SMX monotherapy to complete a 12-month course.

The management of the condition also required balancing infection control against autoimmune hepatitis activity. Azathioprine was held after disseminated nocardiosis was diagnosed, and prednisone was continued at 25 mg/day rather than further tapered during the acute infection-management period. Following immunosuppression modification, liver tests showed a biochemical AIH flare, with AST increasing from 48 U/L to 190 U/L and ALT increasing from 46 U/L to 220 U/L. Despite this biochemical flare, hepatic synthetic function remained preserved, with an INR of 1.1, and total bilirubin continued to improve from 2.9 mg/dL to 1.8 mg/dL. Hepatology recommended continued close monitoring with avoidance of azathioprine re-escalation during uncontrolled disseminated infection. Follow-up liver tests subsequently improved, with an AST 52 U/L and ALT 66 U/L. This course illustrates the competing priorities of reducing immunosuppression during severe opportunistic infections while preventing clinically significant AIH relapse (11).

### Follow-up and outcomes

By hospital day 15, encephalopathy and expressive aphasia had improved as he spoke in short phrases and had recovered his right upper extremity strength. Repeat brain MRI was planned 2 months after antibiotic initiation, with reimaging every 2 months thereafter if abscesses persist. He continued to be afebrile without leukocytosis and was transferred in stable condition to acute inpatient rehabilitation on hospital day 18 after PM&R evaluation. Follow-up brain MRI approximately 1 month after discharge demonstrated interval decrease in size of all bilateral cerebral hemisphere abscesses—with the representative left thalamic lesion decreasing from 2.5 × 2.0 cm to 1.4 × 0.7 cm—along with resolution of internal restricted diffusion, decreased surrounding vasogenic edema, and resolution of the extra-axial collection over the right frontal lobe (Figure 2). The initial TMP-SMX course was complicated by a drug rash, prompting the transition to linezolid. Linezolid was selected because the isolate was susceptible and because it provided an oral agent with high bioavailability and CNS penetration suitable for prolonged therapy after TMP-SMX intolerance (12). Amikacin was avoided for prolonged outpatient therapy because of toxicity and monitoring concerns, while ciprofloxacin resistance and intermediate ceftriaxone/minocycline susceptibility limited those alternatives. Subsequent linezolid-related anemia required monitoring. At 3-month follow-up, the patient had recovered to approximately 80–90% of his pre-illness baseline, with resolution of dysarthria and expressive aphasia, substantial improvement in right-sided motor strength, and tolerance of an oral diet with removal of the PEG tube, though mild short-term memory deficits and residual right upper extremity numbness persisted. At that visit, he was independent in basic activities of daily living and had a modified Rankin Scale score of 2. He remained on intravenous imipenem and oral linezolid, with a planned total treatment duration of 12 months.

**Figure 2:**
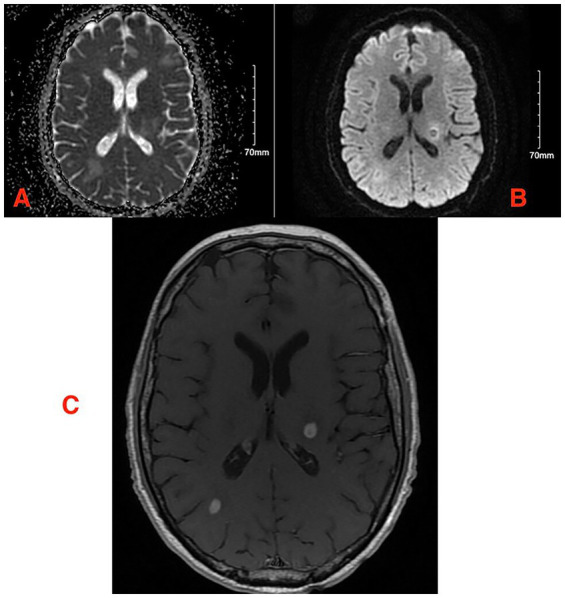
Follow-up brain MRI approximately 1 month after discharge. Representative axial magnetic resonance images, including apparent diffusion coefficient mapping **(A)**, diffusion-weighted imaging **(B)**, and post-contrast T1-weighted imaging **(C)**, demonstrate an interval decrease in the size of the bilateral cerebral hemispheric abscesses, with moderately improved surrounding vasogenic edema. Enhancement is more diffuse, consistent with contraction of the abscess cavities, and there is no longer internal restricted diffusion. The previously seen extra-axial collection over the right frontal lobe has resolved.

## Discussion

This case illustrates disseminated *Nocardia farcinica* presenting with multiple cerebral abscesses and subacute neurologic deterioration in a patient who had recently undergone immunosuppression for autoimmune hepatitis. In hepatology practice, new subacute neurocognitive symptoms during steroid-based induction therapy, particularly during outpatient taper transitions when prophylaxis may be interrupted or incompletely reconciled, should lower the threshold for neuroimaging and tissue diagnosis rather than attribution to steroid effects alone. In retrospect, the patient’s hospitalization 1 month prior for a seizure attributed to hyponatremia—with an unrevealing non-contrast head CT—may have represented an early manifestation of evolving CNS infection not yet apparent on non-contrast imaging, though the clinical attribution to hyponatremia was reasonable at that time.

Immunosuppression that impairs cell-mediated immunity is an established risk factor for nocardiosis (1, 2). In this case, induction therapy for AIH included intravenous methylprednisolone followed by high-dose oral prednisone and azathioprine. Based on medication records, cumulative corticosteroid exposure before nocardiosis diagnosis was approximately 3,300 mg prednisone-equivalent, exceeding thresholds associated with increased infectious complications in an English-language meta-analysis of 71 controlled trials; the geographic distribution of included trials was not reported (13). This degree of corticosteroid exposure, combined with azathioprine, created a clinical context permissive for opportunistic infection. The subsequent need to hold azathioprine and avoid further immunosuppressive escalation during disseminated nocardiosis was clinically necessary but was followed by a biochemical AIH flare, with preserved INR and improving bilirubin, emphasizing the need for close hepatology-infectious disease coordination and serial liver test monitoring. Because CNS involvement is a frequent and clinically consequential manifestation of disseminated nocardiosis, expert reviews recommend brain imaging even when neurologic symptoms are absent (2).

Nocardiosis is most commonly acquired via inhalation, although direct cutaneous inoculation can also occur. In this case, chest/abdomen/pelvis CT did not identify pulmonary nodules, cavitary lesions, focal consolidations, lung abscesses, tree-in-bud opacities, or any other radiographic pulmonary focus of nocardiosis. No cutaneous nodules, ulcers, cellulitis, abscesses, draining wounds, or traumatic inoculation sites were documented. The only microbiologically confirmed extracranial site was the left retroperitoneal collection, which grew the same organism as the cerebral abscess. This supports disseminated disease involving two non-contiguous sites, while the initial portal of entry remains unproven. The absence of radiographically apparent pulmonary disease does not exclude transient, occult, or resolved pulmonary acquisition.

Stereotactic aspiration of the left frontal abscess in this case served both diagnostic and therapeutic purposes. The clinical differential at presentation—which included pyogenic abscess, fungal infection, mycobacterial disease, and metastatic disease—could not be resolved by imaging alone, and tissue sampling was essential for establishing a definitive microbiological diagnosis and guiding targeted therapy. In a systematic review of CNS nocardiosis, patients who underwent surgery combined with antimicrobial therapy had better survival than those treated with antimicrobial alone (OR 2.4, 95% CI 0.99–4.11). Earlier series reported 93% survival with combined medical-surgical management compared with 78% with medical therapy alone (3). These data support early neurosurgical involvement in CNS nocardiosis when feasible, particularly when the microbiologic diagnosis is uncertain.

*N. farcinica* demonstrates a characteristic antimicrobial resistance profile that distinguishes it from other *Nocardia* species and carries direct implications for empiric therapy. In the reported series, *N. farcinica* isolates are resistant to third-generation cephalosporins in approximately 80–100% of cases, including ceftriaxone and cefotaxime, while typically retaining susceptibility to TMP-SMX, imipenem, amikacin, and linezolid (5). In this case, susceptibility testing confirmed intermediate susceptibility to ceftriaxone (MIC 32 μg/mL) and ciprofloxacin resistance, indicating that the initial empiric regimen of vancomycin, ceftriaxone, and metronidazole—appropriate for suspected pyogenic brain abscess—would have been inadequate for the *Nocardia* component. This reinforces the importance of tissue sampling and susceptibility-guided therapy in immunosuppressed patients with CNS lesions. In a large retrospective cohort, *N. farcinica* was independently associated with 1-year mortality, even after adjustment for the extent of infection (14).

CNS nocardiosis carries substantial morbidity and mortality. In a systematic review of 206 patients, the overall case-fatality rate was 22.8%, with immunodeficiency status independently associated with poor outcome (OR 0.32 for good outcome, 95% CI 0.15–0.90) (3), and 1-year all-cause mortality after nocardiosis is approximately 25% (14). Current expert recommendations advise at least 6 months of antimicrobial therapy for most nocardiosis, with longer durations of 12 months or more preferred for CNS and disseminated disease, particularly in immunocompromised hosts (15–17). In the systematic review, the mean duration of therapy was 12 months in survivors with disseminated infection, and treatment for fewer than 6 months was associated with higher mortality and relapse rates (3). Accordingly, our patient was placed on a planned 12-month course. At 3-month follow-up, the patient had achieved substantial clinical and radiographic improvement, though mild cognitive deficits and right upper-extremity numbness persisted.

TMP-SMX chemoprophylaxis, widely used to prevent pneumocystis in immunocompromised patients, has been associated with reduced nocardiosis risk, although that protection is incomplete (6). In this case, disseminated nocardiosis was identified after an estimated 2–4 weeks of interruption of prescribed TMP-SMX prophylaxis during ongoing corticosteroid therapy. This interruption should be interpreted as a potential vulnerability point rather than as proof of causation, because the exact duration could not be definitively reconstructed, and breakthrough nocardiosis can occur despite TMP-SMX prophylaxis. The practical lesson is therefore not that interruption caused the infection, but that prophylaxis reconciliation during outpatient steroid-taper transitions is a modifiable safety step in patients receiving high-dose immunosuppression. Multicenter experience suggests that opportunistic infections in autoimmune hepatitis are uncommon overall, but rare infections can occur in immunosuppressed patients, supporting a broad differential diagnosis when neurologic symptoms develop (18).

The comparative literature highlights both the rarity and heterogeneity of CNS nocardiosis associated with autoimmune hepatitis. Prior AIH-associated CNS nocardiosis cases are summarized in Table 2. Zakaria et al. described a patient receiving azathioprine and prednisolone who developed *N. asteroides* brain abscess complicated by ventriculitis, hydrocephalus, decompressive craniectomy, and severe residual disability (7). Shimada/Yokoe et al. reported *N. farcinica* brain abscess in a woman receiving prednisolone for autoimmune hepatitis, notable for ventricular rupture, absence of pulmonary findings on chest imaging, and discordance between susceptibility testing and clinical response (8). Zintgraff et al. reported *N. farcinica* brain abscess in a patient receiving prednisolone, with both pulmonary and cutaneous findings (9). Pongkaew et al. reported disseminated *N. otitidiscaviarum* with ruptured intracranial mycotic aneurysm in a patient receiving prednisolone and azathioprine for autoimmune hepatitis (10). Compared with these cases, the present report is distinctive for induction-phase AIH immunosuppression, suspected prophylaxis interruption during outpatient steroid taper transition, multiple hemorrhagic cerebral abscesses, culture confirmation from both cerebral and retroperitoneal sites, absence of confirmed pulmonary or cutaneous involvement, and longitudinal radiographic improvement with susceptibility-guided therapy.

**Table 2:** Reported CNS nocardiosis cases associated with autoimmune hepatitis.

Study	AIH therapy/host context	Species	CNS and extracranial involvement	Treatment, outcome, and relevance
Zakaria et al., 2008 (7)	40-year-old man; azathioprine and prednisolone for 1.5 years	*N. asteroides*	Occipital brain abscess with cerebritis, ventriculitis, and hydrocephalus; extracranial disease not emphasized	Aspiration, TMP-SMX then minocycline/meropenem, decompressive craniectomy, and VP shunt; survived with major residual disability
Shimada/Yokoe et al., 2016 (8)	74-year-old woman; prednisolone 16 mg/day; no TMP-SMX prophylaxis	*N. farcinica*	Brain abscess ruptured into the lateral ventricle; no abnormal lung findings on chest X-ray or CT	Surgical drainage, TMP-SMX/amikacin, temporary imipenem-cilastatin/amikacin, then TMP-SMX re-treatment; improved despite susceptibility/clinical-response discordance
Zintgraff et al., 2020 (9)	60-year-old man; prednisolone 10 mg/day	*N. farcinica*	Brain abscess with cavitary left upper-lobe pulmonary lesion and right-hand cutaneous nodule	Aspiration, TMP-SMX plus linezolid, then oral TMP-SMX; abscess decreased, emphasizing species identification and susceptibility testing
Pongkaew et al., 2024 (10)	51-year-old man; prednisolone 30 mg/day and azathioprine 50 mg/day for 7 weeks	*N. otitidiscaviarum*	Ruptured intracranial mycotic aneurysm with intracranial hemorrhage; pulmonary consolidation and disseminated disease	Bifrontal craniectomy, clipping, TMP-SMX, amikacin, and levofloxacin; fatal outcome
Current case	55-year-old man; induction methylprednisolone/prednisone plus azathioprine; TMP-SMX prophylaxis interrupted	*N. farcinica*	Multiple hemorrhagic cerebral abscesses and retroperitoneal abscess; no confirmed pulmonary or cutaneous involvement	Stereotactic aspiration, imipenem-cilastatin plus TMP-SMX, then linezolid after TMP-SMX rash; substantial clinical and MRI improvement at 3 months

AIH, autoimmune hepatitis; CNS, central nervous system; CT, computed tomography; MRI, magnetic resonance imaging; TMP-SMX, trimethoprim-sulfamethoxazole; VP, ventriculoperitoneal.

### Strengths and limitations

This report is strengthened by culture-confirmed *Nocardia farcinica* from cerebral and retroperitoneal sites, susceptibility-guided therapy, neurosurgical and image-guided drainage, and serial clinical/MRI follow-up. Limitations include the single-case design, retrospective reconstruction, uncertain duration of TMP-SMX interruption, inability to infer causality, and an unproven portal of entry; occult or resolved pulmonary acquisition cannot be excluded. The modified Rankin Scale score was assigned based on documented follow-up functional status.

### Take-away lessons

1. In immunosuppressed patients with autoimmune hepatitis, subacute neurologic decline with ring-enhancing lesions should prompt consideration of nocardiosis and early tissue diagnosis.
2. Susceptibility testing is essential for *N. farcinica* CNS infection because resistance can make standard empiric brain-abscess regimens inadequate.
3. Prolonged multidisciplinary treatment is required, while TMP-SMX interruption should be viewed as a reconciliation vulnerability rather than a proof of causation.

## Patient perspective

At 3-month follow-up, the patient described overall recovery to approximately 80–90% of his pre-illness baseline. He reported that the most meaningful improvements were resolution of dysarthria and expressive aphasia and the ability to resume an oral diet after PEG removal. He also noted persistent mild short-term memory difficulty and residual right upper extremity numbness, which remained noticeable in daily life. Functionally, he was independent in basic activities of daily living at 3-month follow-up, corresponding to a modified Rankin Scale score of 2.

## Data Availability

The original contributions presented in the study are included in the article/Supplementary material, further inquiries can be directed to the corresponding author.
