# Supplementary material for: Disseminated Nocardia farcinica with multiple cerebral abscesses in a patient receiving induction immunosuppression for autoimmune hepatitis: case report
Source: Front Med (Lausanne). 2026 Jul 16;13:1825538. doi: 10.3389/fmed.2026.1825538 (PMC13422563; doi:10.3389/fmed.2026.1825538)
Supplement: Supplementary file 1 [file Data_Sheet_1.DOCX]

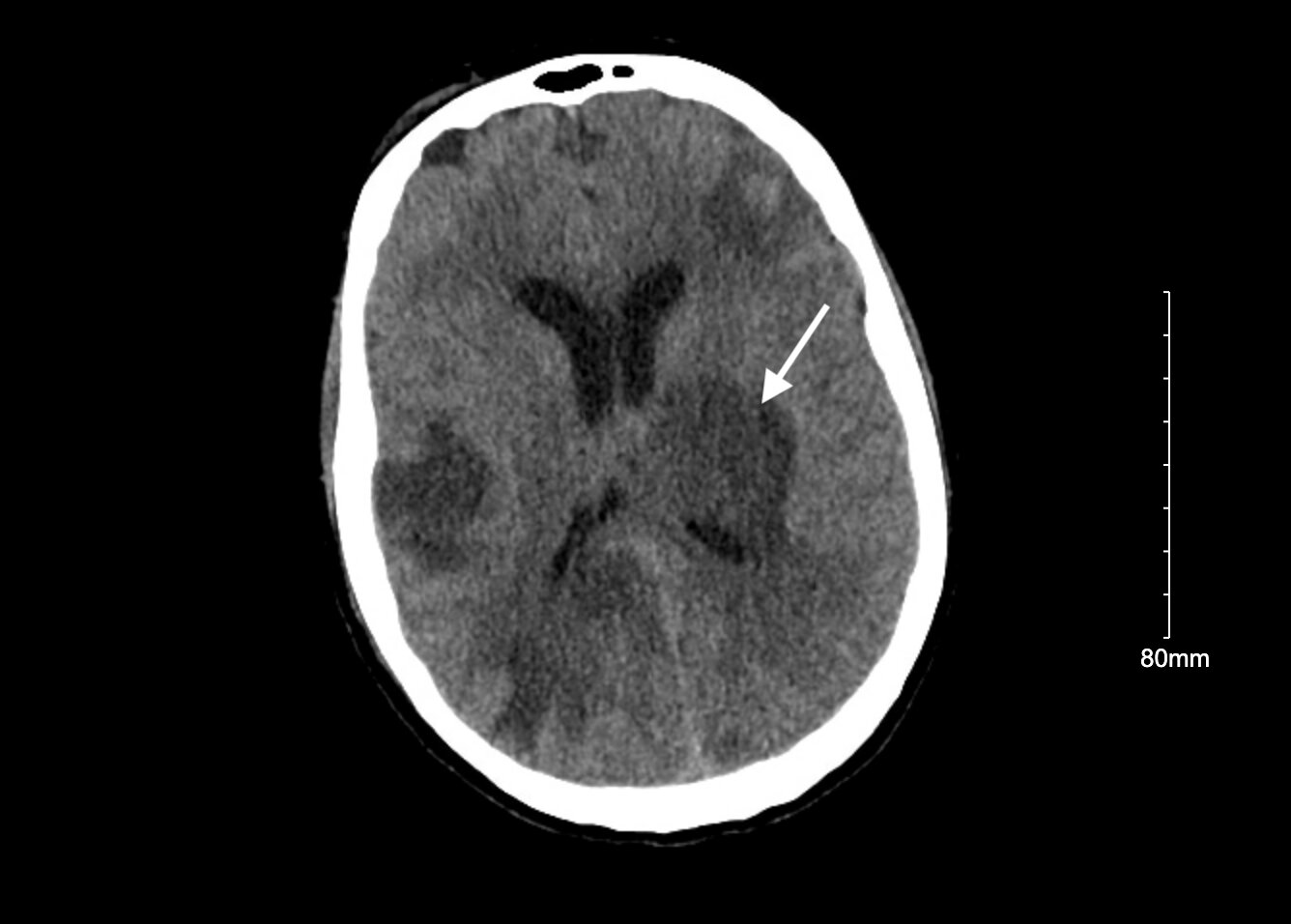
**Supplementary Figure 1. Noncontrast head CT.** Axial noncontrast head computed tomography demonstrating multiple supratentorial hemorrhagic intra-axial lesions with surrounding vasogenic edema, including a dominant left thalamic lesion (arrow). Subsequent brain MRI further characterized these lesions as intracranial abscesses.
