# Supplementary material for: Disseminated Nocardia farcinica with multiple cerebral abscesses in a patient receiving induction immunosuppression for autoimmune hepatitis: case report
Source: Front Med (Lausanne). 2026 Jul 16;13:1825538. doi: 10.3389/fmed.2026.1825538 (PMC13422563; doi:10.3389/fmed.2026.1825538)
Supplement: Supplementary file 2 [file Data_Sheet_2.docx]

**CARE Checklist**

Manuscript: Case Report: Disseminated Nocardia farcinica With Multiple Cerebral Abscesses in a Patient Receiving Induction Immunosuppression for Autoimmune Hepatitis

| **CARE element** | **Locator / reported on** | **How addressed** |
| --- | --- | --- |
| Title | Title | Title identifies the manuscript as a case report and states the area of focus. |
| Keywords | Front matter: Keywords | Key terms provided. |
| Abstract | Abstract | Structured summary includes background, clinical presentation, diagnosis, intervention, and outcome. |
| Introduction | Introduction | Explains why the case is unique, situates it in the literature, and summarizes comparable AIH-associated CNS nocardiosis cases. |
| Patient information | Case Description | De-identified demographics, presentation, relevant history, medications, occupational exposure, and prior interventions. |
| Clinical findings | Case Description; Diagnostic Assessment; Therapeutic Intervention | Neurologic examination, differential leukocyte count, admission liver tests, and subsequent liver-function trends described. |
| Timeline | Table 1: Clinical timeline | Chronologic summary of episode of care, including leukocyte differential, targeted therapy transition, immunosuppression modification, and biochemical AIH flare. |
| Diagnostic assessment | Diagnostic Assessment | Imaging, differential diagnosis, microbiology, susceptibility testing, exclusion of competing diagnoses, and pulmonary/cutaneous extent assessment. |
| Therapeutic intervention | Therapeutic Intervention | Empiric therapy, neurosurgical aspiration/drainage, targeted antimicrobial therapy, PEG placement, treatment plan, and AIH management during infection. |
| Follow-up and outcomes | Follow-up and Outcomes | Clinical course, rehabilitation transfer, follow-up MRI, adverse effects, linezolid transition, 3-month recovery status, and modified Rankin Scale score. |
| Discussion | Discussion; Strengths and Limitations; Take-away Lessons | Interpretation, comparison with AIH-associated CNS nocardiosis literature, clinical implications, prophylaxis limitations, immunosuppression balance, strengths, limitations, and take-away lessons. |
| Patient perspective | Patient Perspective | Patient-reported recovery status, persistent deficits, and functional status at follow-up. |
| Informed consent | Front matter: Signed patient informed consent | Written informed consent obtained. |
